# Staging study of single-channel sleep EEG signals based on data augmentation

**DOI:** 10.3389/fpubh.2022.1038742

**Published:** 2022-11-23

**Authors:** Huang Ling, Yao Luyuan, Li Xinxin, Dong Bingliang

**Affiliations:** ^1^College of Electrical and Information Engineering, Lanzhou University of Technology, Lanzhou, China; ^2^Key Laboratory of Gansu Advanced Control for Industrial Processes, Lanzhou University of Technology, Lanzhou, China; ^3^National Demonstration Center for Experimental Electrical and Control Engineering Education, Lanzhou University of Technology, Lanzhou, China

**Keywords:** EEG, data augmentation, DCGAN, sleep stage, time–frequency analysis

## Abstract

**Introduction:**

Accurate sleep staging is an essential basis for sleep quality assessment and plays an important role in sleep quality research. However, the occupancy of different sleep stages is unbalanced throughout the sleep process, which makes the EEG datasets of different sleep stages have a class imbalance, which will eventually affect the automatic assessment of sleep stages.

**Method:**

In this paper, we propose a Residual Dense Block and Deep Convolutional Generative Adversarial Network (RDB-DCGAN) data augmentation model based on the DCGAN and RDB, which takes two-dimensional continuous wavelet time–frequency maps as input, expands the minority class of sleep EEG data and later performs sleep staging by Convolutional Neural Network (CNN).

**Results and discussion:**

The results of the CNN classification comparison test with the publicly available dataset Sleep-EDF show that the overall sleep staging accuracy of each stage after data augmentation is improved by 6%, especially the N1 stage, which has low classification accuracy due to less original data, also has a significant improvement of 19%. It is fully verified that data augmentation by improving the DCGAN model can effectively improve the classification problem of the class imbalance sleep dataset.

## Introduction

Sleep is of great importance in people's daily life (1), long-term sleep disorders can seriously threaten people's physical and mental health (2). A valid sleep quality assessment method is essential for people to understand their sleep situation and to carry out sleep improvement activities. Sleep staging is an important basis for assessing the quality of sleep and is the first step in diagnosing sleep disorders and helping to improve sleep (3). The electroencephglogram (EEG) signal is widely used in sleep staging as a bioelectrical signal that can directly reflect brain activity. The traditional manual interpretation based on polysomnography can complete the sleep staging well, but it is also easily affected by personal experience to a certain extent and has strong subjectivity, Besides, this manual calibration method is time consuming and labor intensive.

With the introduction of the concept of deep learning by Hinton et al. at the University of Toronto in 2006 (4–6), more and more scholars and research experts tend to use this method that can automatically extract signal features for sleep staging, which has greatly improved the efficiency of sleep staging. However, due to the serious imbalance in the proportion of different sleep stages in the whole sleep process, there is a serious imbalance in the amount of sleep data in the existing sleep EEG dataset, where the N2 stage accounts for 45–55% of the whole sleep time, N3 stage accounts for ~20%, Rapid Eye Movement (REM) stage accounts for ~25%, and the N1 stage accounts for only 2–5% (7). This kind of imbalance greatly affects the accuracy of sleep staging. In the literature (8), the authors obtained an overall classification accuracy of 76% using Convolutional Neural Networks (CNNs) for automatic sleep stage classification but only 60% for the minority class S1 (N1). In the literature (9), the automatic sleep staging method using Convolutional Neural Network—Uni-Directional Long–Short-Term Memory Network (CNN-Uni-LSTM) and Convolutional Neural Network—Bi-Directional Long–Short-Term Memory Network (CNN-Bi-LSTM), respectively, obtained staging accuracy of 80.7% and 82.5%, but only 30.1% and 38.9% for stage N1, respectively. In the literature (10), a depth model classifier was proposed for sleep staging with single-channel EEG signals by combining representation learning (RL) network and Temporal Convolutional Neural Network + Conditional Random Field (TCNN+CRF) and achieved an overall sleep staging accuracy of 81.86% but only 40.2% for stage N1. It has been shown that the imbalance of data classes in all stages of sleep can be effectively improved by data augmentation (11, 12). However, these traditional minority class EEG data augmentation methods, such as time-shifted rolling augmentation (13), overlapping (14), and boundary synthetic minority oversampling algorithm (SMOTE), and its modified model (15), have certain limitations. For example, it is only possible to repeatedly test how many neighbor samples to select according to the specific dataset which results in the blindness of neighbor selection and if the negative class samples are at the distribution edge of the negative class sample set, then the “artificial” samples generated by the neighboring samples and the negative class samples will also be at this edge and will become more and more marginalized which resulting in the limitations of the marginalization of distribution.

Goodfellow et al. (16) proposed a semi-supervised feature learning algorithm based on game scenarios—Generative Adversarial Network (GAN), which provides a more efficient method for data augmentation and brings new ideas for EEG data augmentation. In the literature (17), an artificial EEG signal that is very similar to a single-channel real EEG signal in both the time and frequency domains is stably generated by gradually relaxing the gradient constraints of the Wasserstein GAN (WGAN). However, the instability of GAN networks is highly likely to lead to the phenomenon that the output is not ideal during the training process, and the system is prone to collapse. To address these problems, Radford et al. (18) proposed a Deep Convolutional Generative Adversarial Network (DCGAN), which introduces a supervised learning CNN model into the GAN network to realize the unsupervised learning process of the GAN, generating relatively good quality samples and effectively improving the performance of the GAN. Fahimi et al. (19) proposed a DCGAN-based framework for generating 1D synthetic EEG signals to enhance the training set. Aznan et al. (20) used DCGAN, WGAN, and variational autoencoder to create synthetic EEG data to improve the steady-state visual evoked potential classification task. Choo S et al. (21) proposed a new EEG data augmentation framework using DCGAN to improve the performance of CNN classifiers in motion picture tasks. Xu et al. (22) proposed a DCGAN model to generate artificial EEG data for scaling up stroke datasets, and finally, demonstrated the effectiveness of the generated artificial EEG data. The literature (23) used DCGAN networks to generate synthetic epileptic EEG data in a patient-specific manner thereby improving epilepsy prediction performance. From the above studies, it can be seen that data augmentation models based on DCGANs networks have been widely used in EEG with relevant results, but it was found that the following problems still exist in the application of sleep EEG:

- For the more complex sleep EEG signals, traditional GANs and DCGANs have limited ability to extract their one-dimensional data features, and it is difficult to guarantee the quality of EEG signal generation at each stage.
- Traditional DCGANs models suffer from model instability, inability to extract deep features of images, and lack of details in the generated images during the training process of image data.

To address these problems, this paper proposes a sleep EEG data augmentation model RDB-DCGAN based on Residual Dense Block (RDB) and DCGAN. First, the original 1D EEG signal is converted into the 2D time–frequency map for data augmentation, and the Residual Dense Network (RDN) formed by the RDB is fused in the generator to strengthen feature propagation and alleviate the problem of gradient disappearance. The discriminator loss function of the RDB-DCGAN network is improved by combining the perceptual loss with the cross-entropy loss function and adding the gradient penalty to obtain more image details and make the network training more stable. Finally, CNN is used to classify the sleep EEG signals after data augmentation.

## GAN and DCGAN theory

**Figure 1:**
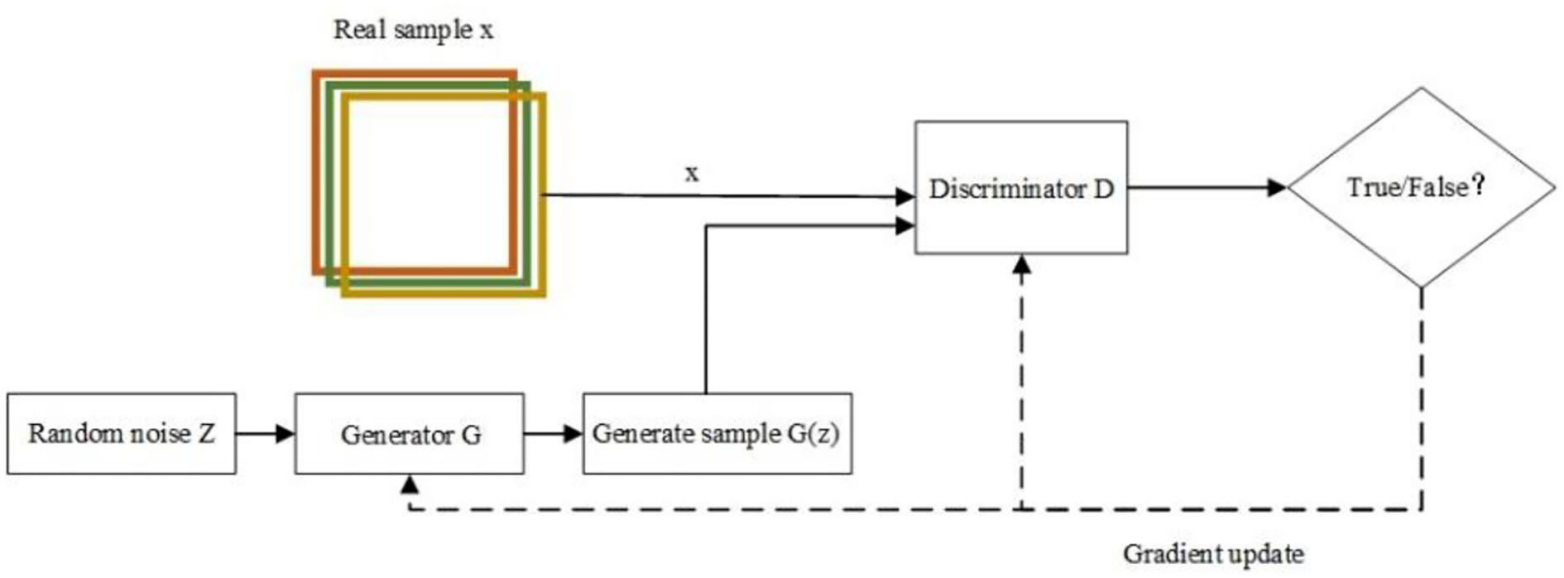
A schematic diagram of the GAN network.

Generative Adversarial Network consists of two parts, a generator G and a discriminator D. The generator G uses the input noise Z to generate generative samples G(Z) that can deceive the discriminator through continuous feature learning, and the discriminator D is used to discriminate whether the input samples are real samples x or generative samples G(Z) and then reverse the parameters of the generator to make the generator generate more realistic samples. In this process, the generator (G) and the discriminator (D) are trained alternately and continuously confronted, and finally, the generated samples G(z) generated by the generator G are indistinguishable from the real data (24, 25). The training process of GAN is shown in Figure 1.

Deep Convolutional Generative Adversarial Network improves the generator and discriminator of the original GAN network into a deep convolutional network structure specifically for generating image samples. In DCGAN, the discriminator retains the overall architecture of CNN, while the generator replaces the convolutional layer with a Convolution Transpose layer. Finally, the DCGAN generator uses the ReLU activation function for all layers except the output layer, which uses the Tanh activation function, while the discriminator uses the Sigmoid activation function for the output layer to prevent gradient sparsity and the Leaky ReLU activation function for all other layers.

## Data augmentation model based on improved DCGAN network

**Figure 2:**
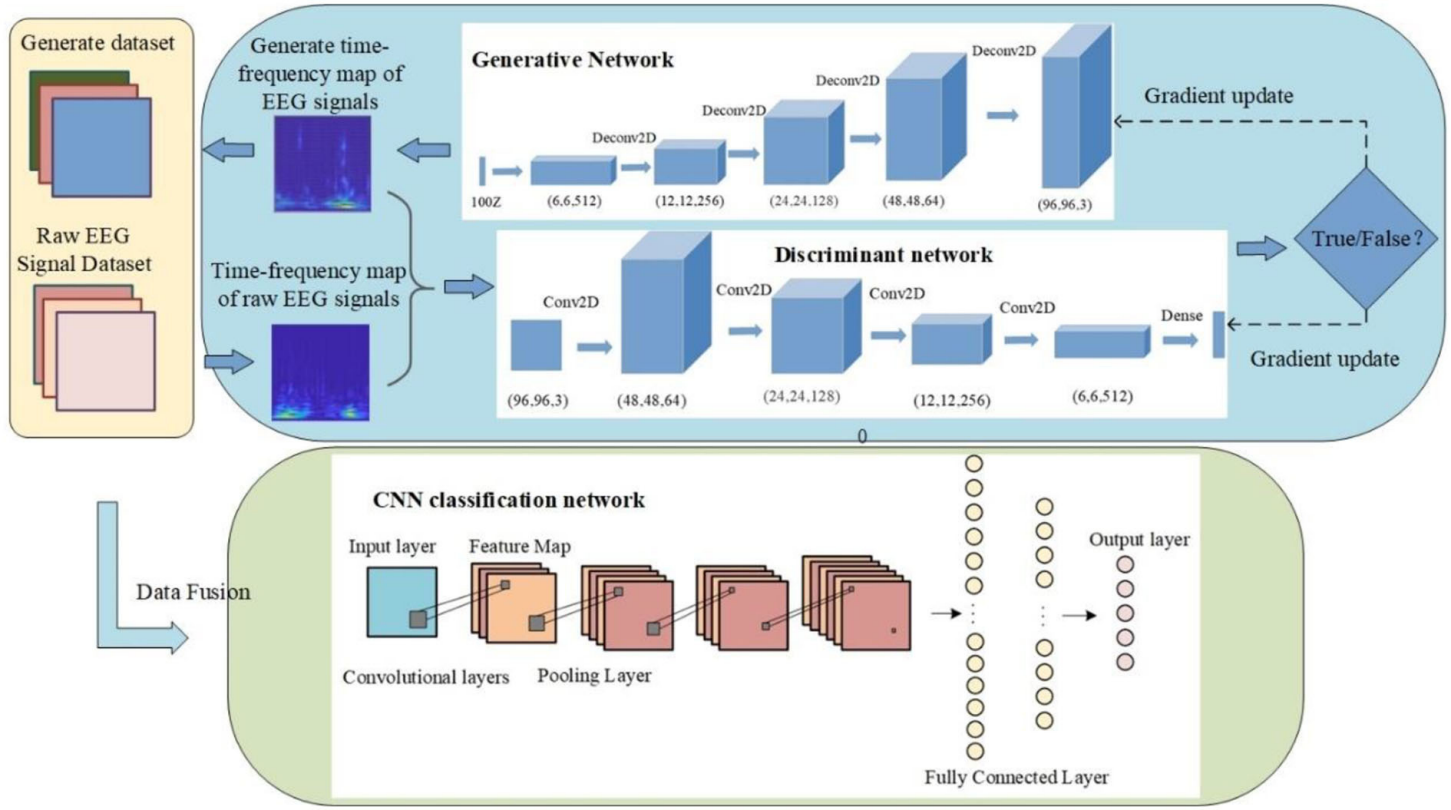
The general framework of the model.

The overall network model in this paper is divided into two main parts: the improved DCGAN sleep EEG data augmentation model and the CNN sleep EEG staging model. The design of the improved DCGAN data augmentation model is mainly divided into three parts as follows: generator, discriminator, and loss function. The model adds an RDN formed by RDB to strengthen the propagation between features, obtain more global dependencies within the features, generate higher-quality images, and finally, form an RDB-DCGAN data augmentation model. The loss function part combines perceptual loss with cross-entropy and adds a gradient penalty to make the network training more stable. In addition, compared with the traditional method of feature extraction for classification, CNN has the powerful feature extraction ability for image data, which can better achieve the classification of different sleep stages. The samples of different sleep stages are expanded by the improved DCGAN network, and the original samples are mixed with the generated samples to form a new dataset with the same number of samples of each type. The new dataset is used to train the CNN for sleep staging with class imbalance. The general framework of the model is shown in Figure 2.

### Generator improvement

**Figure 3:**
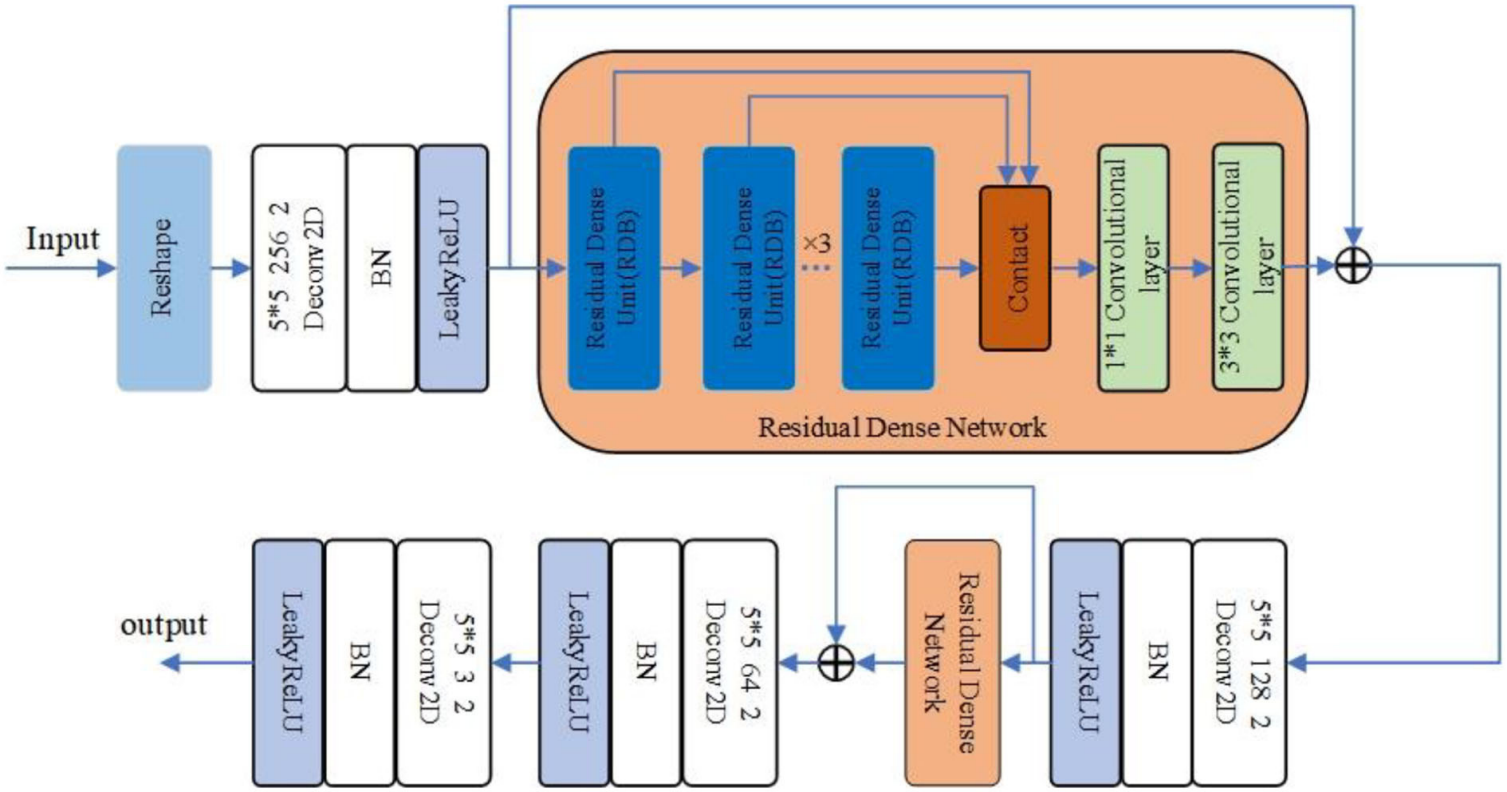
RDB-DCGAN generator model.

**Figure 4:**
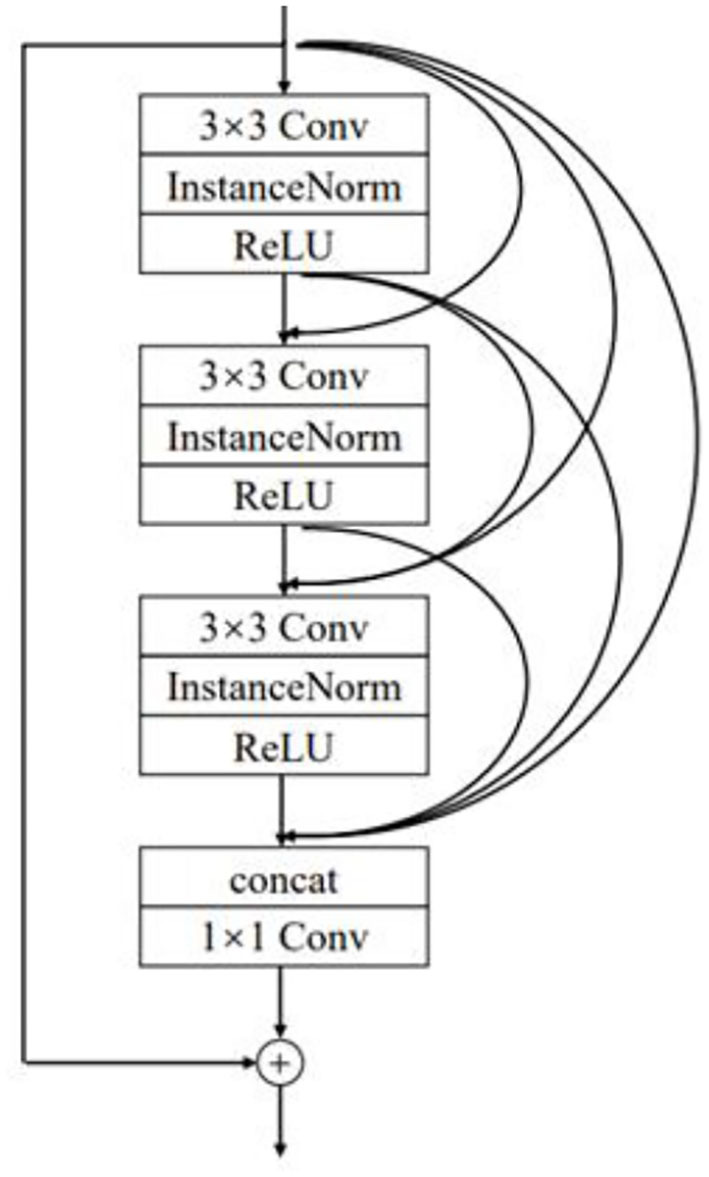
Schematic diagram of residual dense cell structure.

In the improved DCGAN network, the generator is mainly used to generate a minority class of sleep EEG time–frequency maps. In this paper, the generator adopts the structure of transposed convolution, which consists of four layers of transposed convolution operations, and the RDN consisting of six RDBs is added between the first and second layers of transposed convolution and between the second and third layers of transposed convolution. Additionally, after the RDN, the input and output features are fused as the new output. The combination of shallow features and different layered features extracted from each unit of the RDN through the input–output fusion provides richer image details, which is helpful to generate more realistic sleep EEG time–frequency maps. The improved RDB-DCGAN generator model is shown in Figure 3. Among them, the RDB is a combination of both the residual block and the dense block, which mainly contains two parts: the dense connection and local feature fusion. In the RDB (structure as shown in Figure 4) structure, two layers are directly connected, dense connection can well strengthen the propagation between features, reduce the number of parameters, and better extract the deep features in the image, and the residual structure can effectively alleviate the problem of gradient disappearance with the deepening of the network layers, making the network more stable; in addition, because the cumulative splicing of RDB will lead to too many feature layers, so by the 1^*^1 convolutional layers after dense concatenation is used for the fusion of local features and play the role of dimensionality reduction.

The generator of the RDB-DCGAN model reshapes a 100-dimensional noise vector into (6,6,512) feature maps using the reshape function; after a two-dimensional transpose convolution operation for shallow feature extraction, and converts the output dimension to (12,12,256); after passing through six RDBs in the RDN, and the output of RDB will be combined by stitching and feature fusion using a 1^*^1 convolutional layer, after which the dense feature fusion is completed by adding the results of the first convolution through a convolutional layer with the first convolution, at this time, the input and output image dimensions are the same, then after a transpose convolution and again into the RDB, and finally, through two transpose convolutions and using the Tanh activation function to output it as the required image sample size (96,96,3). The convolution kernels of all transposed convolution layers in the generated network are set as small as 3^*^3 and the step size is set as 2.

### Discriminator design

**Figure 5:**
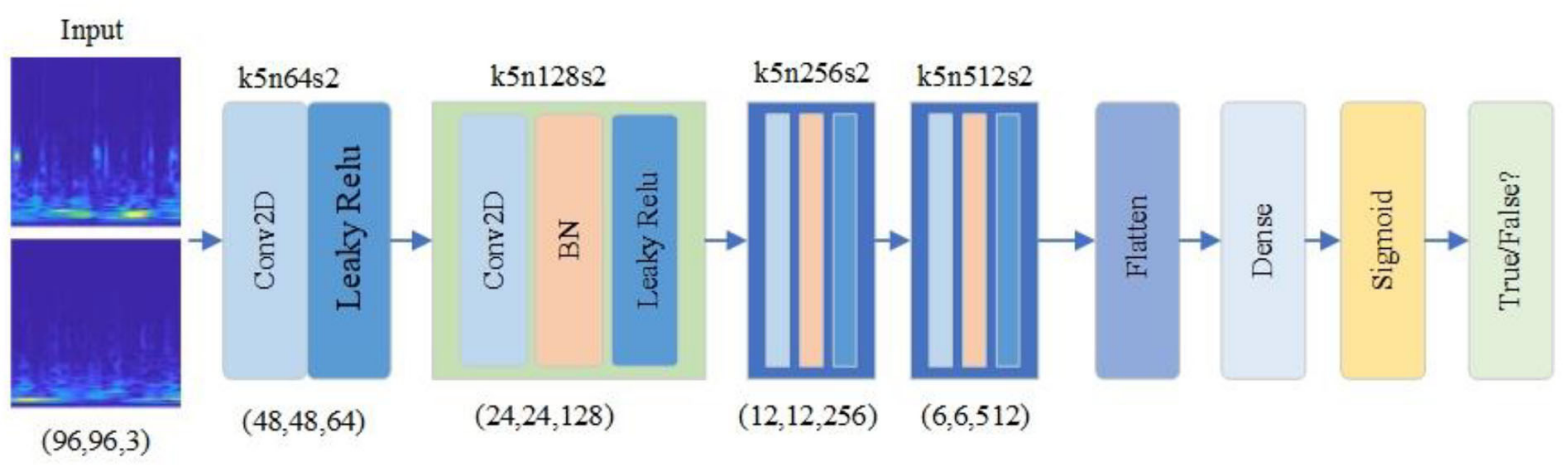
RDCGAN discriminator model.

The improved RDB-DCGAN discriminator model is shown in Figure 5. The discriminator converts the image of size (96,96,3) into a scalar through four layers of convolutional operations, converts the sample data of dimension (96,96,3) into dimension (6,6,512) by four convolutional kernels of size 5^*^5 and two-dimensional convolutional operations of step size 2, converts the multi-dimensional input into one-dimensional through the Flatten layer, and finally, converts the output estimate through the fully connected layer to determine whether the given image is true or false, and the output value is 0 or 1.

In the actual training process of the RDB-DCGAN data augmentation model, the training of the discriminator and generator is carried out alternately, and the original EEG signal time–frequency map and the generated EEG signal time–frequency map are put into D for training to maximize the discriminative accuracy of D; the generator G is optimized, and the 100-dimensional noise vector is put into G for training to generate new EEG signals to minimize the discriminative accuracy. The alternating training cycle continues, and the generated data of the generator are so close to the real data that the discriminator cannot accurately identify the real data and the generated data to reach the Nash equilibrium, and the gradient of the generator and discriminator is updated through continuous training to finally output high-quality EEG signal maps.

### Loss function design

To further improve the quality of images generated by the RDB-DCGAN network, this paper improves the original loss function of RDB-DCGAN by combining the content loss function and the adversarial loss function to further optimize the network model.

#### Content loss function

Perceptual loss is selected as the content loss function. Perceptual loss is to compare the features obtained by convolving the real image with the features obtained by convolving the generated image to make the generated image more semantically similar to the target image, thus enhancing the image details and generating a more realistic image. Since perceptual loss uses neural networks to extract deeper feature maps, deeper network layers are more conducive to extracting deeper semantic information, so VGG19 (26) is used. The deep convolutional layers of the pre-training model are used to extract the features of the generated and original maps separately, and then the Euclidean distance between the two maps is calculated with the following equation:


(1) $$\begin{array}{l} L_{VGG}\left(G\right)=\frac{1}{w_{j}h_{j}d_{j}}\|VGG\left(G\left(z\right)\right)-VGG\left(x\right){\|}_{F}^{2} \end{array}$$

where *w*_*j*_*h*_*j*_*d*_*j*_ represents the width, height, and depth of the *i*-th feature space, *G*(*z*) is the generated image, and *x* is the real image, respectively.

#### Contrast loss function

The cross-entropy loss function is commonly used in DCGAN networks to describe the degree of difference between two different probability distributions. During the training of the network, the actual is *G*(*z*) should be as close as possible to the data distribution of the real image *P*_*data*_(*x*). Based on the cross-entropy loss function, the loss function can be constructed as follows:


(2) $$\begin{array}{l} V\left(D,G\right)=E_{x\sim P_{\left(data\left(x\right)\right)}}\left[InD\left(x\right)\right]+E_{z\sim P_{z}\left(z\right)}\left[In\left(1-D\left(G\left(z\right)\right)\right)\right] \end{array}$$

where *E*_*x*~_*P*__(*data*(*x*))__is the real sample obtained from the training data *x*; *E*_*z*~_*P*__*z*_(*z*)_ is the sample obtained in the noise distribution; *D*(*x*) represents the probability of *D* judging whether the real images are real or not, so for *D*, the larger this value is, the better; *D*(*G*(*z*)) is the probability of *D* judging whether the images generated by *G* are real or not, so *G* wants *D*(*G*(*z*)) to be as large as possible, i.e., the smaller *E*_*z*~_*P*__*z*_(*z*)_[*In*(1 − *D*(*G*(*z*)))]is, the better. From this, the objective function *L*_*a*_is obtained as follows:


(3) $$\begin{array}{l} \begin{array}{lll} L_{a} & = & V(D,G)=E_{x\sim P_{\left(data(x)\right)}}[InD(x)] \end{array}\\ \begin{array}{l} \;+E_{z\sim P_{z}(z)}[In(1-D(G(z)))]. \end{array}\; \end{array}$$

The final loss function of the RDB-DCGAN network is the weighted sum of the adversarial loss function and the content loss function, which are jointly used to optimize the data augmentation network model, i.e.,


(4) $$\begin{array}{l} L=L_{a}+\lambda_{1}L_{VGG}\left(G\right) \end{array}$$

where λ_1_ is empirically set to 0.1.

### Classification model

**Figure 6:**
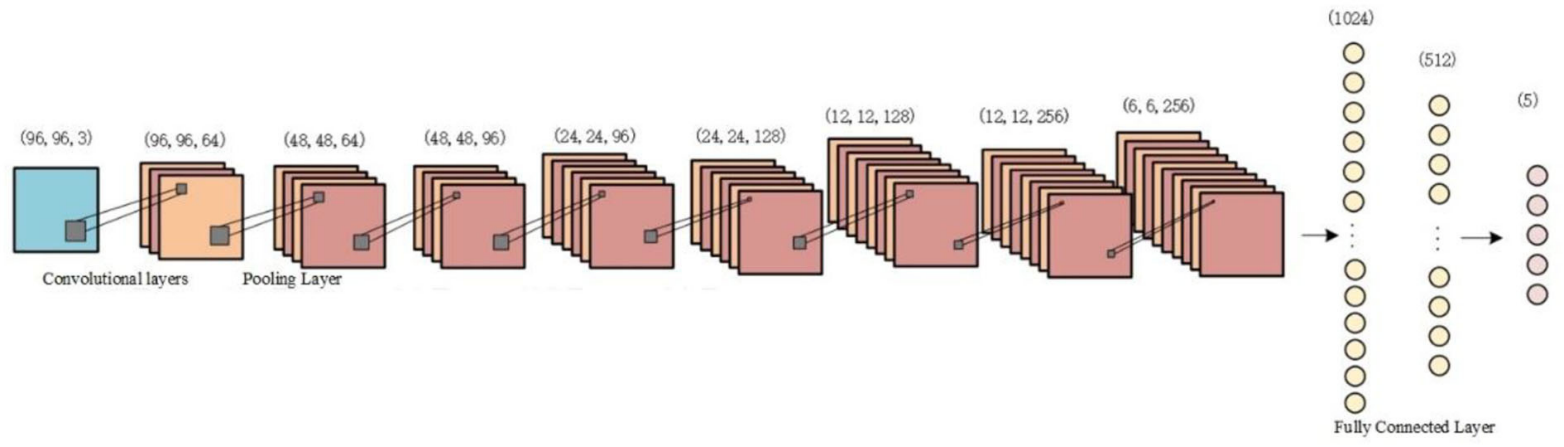
Convolutional neural network (CNN) model structure diagram.

In this paper, a classification model with a typical convolutional neural network architecture is used to verify the impact on classification accuracy before and after data augmentation. The CNN classifier used consists of four convolutional layers, three fully connected layers, and a pooling layer is added between each convolutional layer. A fused time–frequency map of the sleep EEG signal is used as the input to the CNN. The two-dimensional time–frequency image is convolved, pooled, and fully connected, and the output is staged into five categories of sleep EEG signals. The structure of the CNN model is shown in Figure 6.

## Data processing and analysis

**Figure 7:**
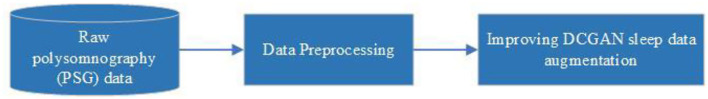
Overall experimental flow.

In this experiment, the Polysomnography (PSG) data of a total of 40 individuals from the SC subset of Sleep-EDF Database Expanded were used to verify that the proposed improved RDB-DCGAN model could effectively improve the impact of the data class imbalance problem on sleep staging. The overall experimental process is shown in Figure 7. First, the sleep EEG data from the original polysomnography database were preprocessed, and the preprocessed sleep EEG data were fed into the improved DCGAN network to realize the data augmentation of EEG signals, and finally, the CNN network was used to complete the staging of sleep EEG signals.

### Data pre-processing

This paper was studied using the Sleep-EDF Database Expanded (27) public sleep EEG database on the PhysioNet system, which contains 2 days of PSG data from 197 subjects, divided into two subsets, SC and ST, where the SC subset is from the healthy population and the ST subset was having mild difficulty in sleeping. In the PSG recorded signals, the EEG signals were taken from Fpz-Cz and Pz-Oz electrode locations, respectively, with a sampling rate of 100 Hz, and the data were sliced by 30 s. The sleep stages of each segment according to the R&K criteria (28) were divided into WAKE, N1, N2, N3, N4, REM, MOVEMENT, and UNKNOWN.

To ensure the consistency of the research data and exclude the influence of sleep difficulties on the research, in this experiment, we used the data of 40 people in Fpz-Cz leads in the SC dataset for the experimental analysis (from SC4001 to SC4211, excluding the discontinuous SC4152 and SC4172 datasets). According to the AASM judgment rule (29), the N3 and N4 periods were combined into the N3 stage data; MOVEMENT and UNKNOWN data were excluded; the sleep EEG signals from before sleep to 15 min after waking were intercepted; the intercepted signals were filtered through a Butterworth eighth-order low-pass filter with a cut-off frequency of 35 Hz; and noise reduction was performed after removing industrial frequency interference. The processed EEG signal is transformed from a 1D signal to a 2D signal by wavelet time–frequency transform to complete the data pre-processing. After data pre-processing, the signal is sliced every 30 s into the corresponding sleep stage to form the training dataset for the corresponding stage.

Table 1 shows the data volume of each sleep stage in the experimental dataset, where stage N1 only accounts for 8% of the total data volume, which shows that the dataset is a class imbalance dataset. Because of the lower data volume of the N1 stage, the accuracy of sleep staging in the N1 class during sleep staging is low compared with other classes. In this paper, the data are expanded by the improved DCGAN data augmentation method to obtain the same number of five-class samples to alleviate the class imbalance in the original dataset, which effectively improves the staging accuracy of sleep stages with lower data volume.

**Table 1:** Amount of data for each sleep stage in the experimental data.

**Sleep period**	**Wake**	**N1**	**N2**	**N3(N4)**	**REM**
Quantity(pcs)	5,037	2,967	15,204	5,390	7,795
Percentage of	14%	8%	42%	15%	21%

### Improved DCGAN data augmentation

The number of original datasets was first expanded using the further improved RDB-DCGAN network for data augmentation. To verify the effectiveness of the proposed improved DCGAN data augmentation method for sleep EEG signal image sample generation, this experiment compares the samples generated by the RDB-DCGAN network, the RDB-DCGAN network with altered loss, and the original DCGAN network after data augmentation. The deep learning framework used in the experiments is Keras, and the hardware platform used is Intel(R) Core(TM) i7-10875H CPU @ 2.30GHz and NVIDIA GeForce RTX 2060Ti GPU.

**Figure 8:**
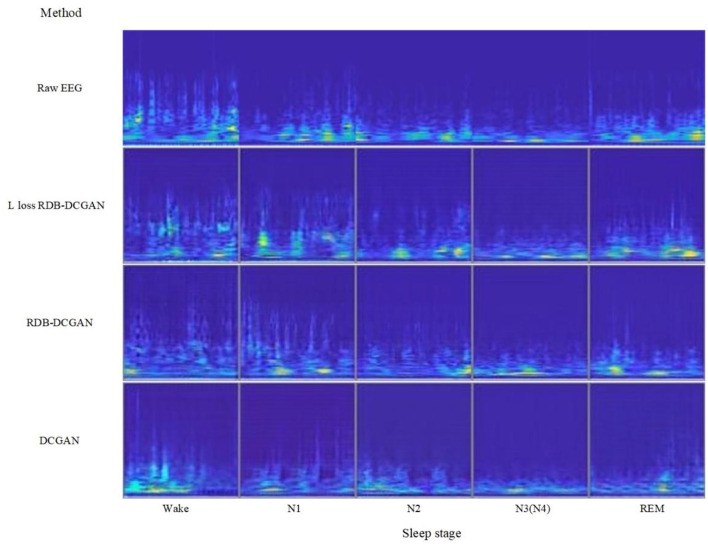
Comparison of the original image and generated image.

The RDB-DCGAN network and DCGAN network models use cross-entropy loss function for the generator and discriminator. The RDB-DCGAN network with modified loss uses the improved loss function, the optimizer is Adam, the discriminator learning rate is set to 0.0002, the generator learning rate is set to 0.0005, the alpha in LeakyReLU is set to 0.2, the momentum in batch normalization is set to 0.9, the number of network iterations is 25000, and the batch size is selected as 64. Raw EEG signal, DCGAN generated EEG signal, RDB-DCGAN, and RDB-DCGAN network with change loss generated EEG signal are compared as shown in Figure 8.

### Improved DCGAN generation data analysis

In this paper, the Fréchet Inception Distance (FID) is used as an objective evaluation index for the EEG signal time–frequency maps generated by each network, and the FID is a good measure of the similarity between the generated images and the real images. FID is calculated as follows:


(5) $$FID\left(x,g\right)={\left\|u_{x}-u_{g}\right\|}_{2}^{2}+Tr\left(\sum_{}^{}x+\sum_{}^{}g-2{\left(\sum_{}^{}x\sum_{}^{}g\right)}^{\frac{1}{2}}\right)$$

where (*u*_*x*_, ∑ *x*) and (*u*_*g*_, ∑ *g*) are the mean and covariance of the true data distribution and the sample data distribution, respectively.

The FID evaluation index is principled and comprehensive, which can accurately reflect the similarity between the generated samples and the real samples, and the smaller the value, the higher the similarity between the samples and the better the generation effect. The FID values of the improved DCGAN are significantly reduced compared with DCGAN on these five classes of the same dataset, and the specific results are shown in Table 2, and the FID values are reduced by 47.3, 51.7, 34.8, 49.8, and 41.8%, respectively.

**Table 2:** Comparison of FID values of data generated by different models.

**Models**	**FID value**				
	**Wake**	**N1**	**N2**	**N3 (N4)**	**REM**
DCGAN	160.7	165.4	162.9	185.0	166.1
RDB-DCGAN	125.0	119.8	120.8	136.7	125.0
L loss RDB-DCGAN	113.4	113.7	128.1	135.2	124.3

## Comparative analysis of classification before and after data augmentation

To further illustrate that data augmentation can effectively improve the problem of low accuracy of sleep first stage classification (N1) caused by class imbalance. The data-enhanced samples were tested for classification with the original samples using CNN. Both the original sleep EEG signal dataset and the data-enhanced sleep EEG signal dataset were divided into training and test sets in the ratio of 7:3 for classification recognition. The initial learning rate of the designed CNN network structure is 0.00005, and the learning rate gradually decreases according to the step size. At the same time, the Adam optimizer and the loss function of cross entropy are used for training. In addition, dropout is set to 0.5.

Use CNN to classify the data before and after data augmentation. Six simulations were performed in this test, and the average of the sleep staging accuracy of each stage, the average of the overall classification accuracy, and the standard deviation were obtained as shown in Table 3.

**Table 3:** Classification accuracy of each stage before and after data augmentation.

**Training samples**	**Sleep Stage**						
	**Wake**	**N1**	**N2**	**N3 (N4)**	**REM**	**Mean**	**Standard Deviation**
Raw data	72.1%	42.6%	73.6%	76.6%	72.3%	69%	0.005
After data augmentation	74.5%	63.2%	80.7%	85.2%	75.2%	75%	0.006

As can be seen from Table 3, after the RDB-DCGAN data augmentation, the staging accuracy of each stage has been improved, and the overall recognition accuracy has increased by 6%, especially the classification accuracy of the N1 stage has been significantly increased by ~19%. Through the calculation of standard deviation, we can also see that the model achieves better classification stability, which further illustrates the effectiveness of data augmentation for classification.

To further demonstrate the effect of the improved DCGAN data augmentation and to comprehensively evaluate the performance of the model, the classification performance was evaluated using a confusion matrix, Precision (Pre), Recall (Re), and F1 score (F1), and each criterion was calculated as follows:


(6) $$\begin{array}{l} Pre=\frac{TP}{TP+FP} \end{array}$$

(7) $$\begin{array}{l} Re=\frac{TP}{TP+FN} \end{array}$$

(8) $$\begin{array}{l} F1=\frac{2\times Pre\times Re}{Pre+Re} \end{array}$$

(9) $$\begin{array}{l} Acc=\frac{\left(TP+TN\right)}{\left(TP+TN+FP+FN\;\right)}. \end{array}$$

Among them, *TP* is predicted by the model as a positive-positive sample, *TN* is predicted by the model as a negative–negative sample, *FP* is predicted by the model as a positive–negative sample, and *FN* is predicted by the model as a negative–positive sample.

The confusion matrix generated before and after data augmentation is shown in Tables 4, 5.

**Table 4:** Confusion matrix for each stage before data augmentation.

	**Predicted**					**Per-class metrics**		
	**Wake**	**N1**	**N2**	**N3 (N4)**	**REM**	**Pre**	**Re**	**F1**
Wake	**0.72**	0.06	0.06	0.03	0.12	**0.72**	**0.72**	**0.72**
N1	0.13	**0.44**	0.14	0.02	0.28	**0.65**	**0.44**	**0.52**
N2	0.03	0.02	**0.73**	0.11	0.11	**0.73**	**0.73**	**0.73**
N3(N4)	0.03	0.00	0.17	**0.77**	0.03	**0.72**	**0.77**	**0.74**
REM	0.05	0.06	0.13	0.02	**0.74**	**0.65**	**0.74**	**0.69**

The bold values in the Predicted section represent the classification accuracy of each stage.

**Table 5:** Confusion matrix for each stage after L loss RDB- DCGAN data augmentation.

	**Predicted**					**Per-class metrics**		
	**Wake**	**N1**	**N2**	**N3 (N4)**	**REM**	**Pre**	**Re**	**F1**
Wake	**0.73**	0.12	0.04	0.03	0.08	**0.80**	**0.74**	**0.74**
N1	0.12	**0.63**	0.06	0.03	0.16	**0.74**	**0.64**	**0.69**
N2	0.03	0.01	**0.81**	0.07	0.09	**0.76**	**0.80**	**0.77**
N3(N4)	0.03	0.02	0.06	**0.86**	0.03	**0.83**	**0.84**	**0.87**
REM	0.05	0.06	0.09	0.04	**0.76**	**0.69**	**0.79**	**0.71**

The bold values in the Predicted section represent the classification accuracy of each stage.

Each column of the confusion matrix represents the sample situation predicted by the model and each row of the matrix represents the true situation of the sample. As can be seen from the confusion matrix before and after data augmentation in Tables 4, 5, the staging accuracy of the original EEG signals Wake, N1, N2, N3(N4), and REM are 72, 44, 73, 77, and 74%, respectively, and the staging accuracy of the EEG signals Wake, N1, N2, N3(N4), and REM after data augmentation are 73, 63, 81, 86, and 76%, respectively. Compared with the classification process of the original data, a large number of N1 data were wrongly assigned to the Wake, N2, and N4 stages, and the accuracy of the N1 sleep stage increased significantly after data augmentation. In addition, it can also be seen from Table 1 that the classification accuracy of the Wake, N3, and N4 sleep stages with a small proportion of original data increased after data augmentation, but the classification accuracy of the Wake and REM stages did not increase significantly. We can also see from Tables 4, 5 that the recall of a few classes of N1 has increased from 0.44 to 0.64, and the F1 value has increased from 0.52 to 0.69, both of which have improved more significantly, while the Pre, Re, and F1 values of the remaining sleep stages have also increased, thus verifying the effectiveness of the improved RDB-DCGAN algorithm.

In Table 6, the data in Per-class Performance are the staging accuracy of each stage, numbers in bold indicate the best classification method for each sleep stage. Compared with Li et al. (9) and Khalili et al. (10), although the authors use a more optimized CNN algorithm for sleep staging, we can be seen that the ideal classification effect is not achieved in the N1 stage. To further illustrate the contribution of data augmentation to improve sleep staging accuracy and ensure the validity of the comparison, we compare the results with other papers that use sleep EEG time–frequency maps as input to CNN classifiers for sleep staging (8, 30–32). Compared with the sleep staging results of other papers, the data augmentation method proposed in this paper achieves the best sleep staging accuracy in the N1 stage with the least amount of data. In addition, N2, N3, and REM also achieved good performance. However, the results of the Wake stage are not ideal. Combined with Jadhav et al. (31) and Wei et al. (32) which have higher classification accuracy in the Wake stage, both have more data volume, especially for Wei et al. (32), where the Wake stage accounts for only 28% of the total data volume, which is also advantageous for CNN classification. Second, the design of the CNN network structure may also cause differences in results, so the classification accuracy of the Wake stage needs to be further discussed.

**Table 6:** Comparison of classification results after data augmentation using the RDB-DCGAN model with other classification results.

**Author**	**EEG Channel**	**Per-class Performance**					**Overall Performance**
		**Wake**	**N1**	**N2**	**N3**	**REM**	**Acc**
Tsinalis et al. (30)	Fpz-Cz	70.0	60.0	73.0	**91.0**	74.0	74.8
Tsinalis et al. (8)	Fpz-Cz	81.6	60.0	78.0	89.0	80.4	78.9
Li Q et al. (9)	Pz-Oz	87.7	38.9	88.3	85.6	78.5	82.5
Khalili E et al. (10)	Fpz-Cz	**93.0**	40.2	86.8	73.3	81.5	81.9
Jadhav et al. (31)	—	90.2	27.6	**92.2**	74.0	**86.0**	83.3
Wei L J (32)	Fpz-Cz	92.9	34.9	84.1	83.0	74.7	82.7
Our model	Fpz-Cz	73.0	**63.0**	81.0	86.0	76.0	76.0

## Discussion and conclusion

In this paper, we designed a sleep EEG data augmentation model based on the improved DCGAN network, and in our experiments, we found that:

- According to Figure 8, we can see that through the continuous improvement of the data augmentation network, we have generated a sleep EEG time–frequency map that looks very good to the naked eye, it has also achieved lower FID value through the continuous update of the structure objectively. According to Tables 2–5, we can also see that for the N1 and N3 stages where the quality of the generated image is more improved, the classification accuracy has also achieved a higher growth value. Therefore, it can be concluded that if we can obtain a better quality time–frequency map of sleep stages during data augmentation, it will be of great help to further improve the quality of sleep stages. But at the same time, we found that the Wake stage with a lower FID value (meaning that there are more similar samples in the arousal state) did not achieve a significant improvement after data augmentation. We analyze that the reason may be that due to the similarity of the features of Wake and the features of the N1 stage and the limitation of the CNN network, we cannot further extract deeper features to classify these two stages more accurately (the CNN classifier can be seen in Table 6 misclassified a lot of Wake stage data to N1 stage), so resulted in lower Wake stage growth.
- Second, to illustrate that our method contributes to the classification of class-imbalanced EEG datasets, we use the basic CNN network for classification training and compare the results obtained before data augmentation at each stage of sleep. It has a good classification effect, but the classification accuracy of the Wake stage and REM stage has a small increase. On the one hand, it is also because when the original data were obtained, we intercepted the awake data before going to bed and 15 min after waking up, so the Wake stage has no advantage over the original data. On the other hand, due to the similarity between N1 and REM, as well as N2 and REM (the feature similarity between sleep periods leads to similar EEG signals); more N1 and N2 classes are misclassified as REM classes.
- In addition, it can be seen from Table 6 that through the data augmentation method in this paper, we have achieved superior classification results compared to other CNN classifications in the N1 stage, and it further verifies that the two-dimensional time–frequency map generated by the data augmentation method proposed in this paper brings advantages to sleep staging.

Finally, the use of EEG time–frequency maps for data expansion by image data augmentation also provides a new idea worth exploring for sleep staging. It should also be noted that this paper only discusses the EEG time–frequency map after wavelet transform, and further in-depth research will be done in the future from the perspective of more input features, and more in-depth classification methods will be used to verify the validity and applicability of this paper from more perspectives.

## Data availability statement

Publicly available datasets were analyzed in this study. This data can be found here: https://www.physionet.org/content/sleep-edfx/1.0.0/, Sleep-EDF Database Expanded.

## Ethics statement

Ethical review and approval was not required for the study on human participants in accordance with the local legislation and institutional requirements. Written informed consent for participation was not required for this study in accordance with the national legislation and the institutional requirements. Written informed consent was not obtained from the individual(s) for the publication of any potentially identifiable images or data included in this article.

## Author contributions

HL and YL provided the overall conception and design of the study. YL wrote the manuscript portion of the paper. LX preprocessed the paper data. DB provided comments on revisions to the paper manuscript and oversaw the study. All authors contributed to the article and approved the submitted version.

## Funding

This study was supported by the Science and Technology Key Research and Development Projects in Gansu Province, Ultra-wideband Radar Life Detection System and Key Technology Research, 20YF3GA018.

## Conflict of interest

The authors declare that the research was conducted in the absence of any commercial or financial relationships that could be construed as a potential conflict of interest.

## Publisher's note

All claims expressed in this article are solely those of the authors and do not necessarily represent those of their affiliated organizations, or those of the publisher, the editors and the reviewers. Any product that may be evaluated in this article, or claim that may be made by its manufacturer, is not guaranteed or endorsed by the publisher.
